# The effects of mindfulness meditation training on musical aesthetic emotion processing in Chinese pop music: an empirical study of musically trained individuals

**DOI:** 10.3389/fpsyg.2025.1685030

**Published:** 2025-12-08

**Authors:** Xuanlin He, Qiao Zheng, Haijiao Li

**Affiliations:** Music College, Sichuan Normal University, Chengdu, China

**Keywords:** musical aesthetic emotion processing (MAEP), mindfulness meditation, Chinese popular music, aesthetic attitude, aesthetic judgment, emotional experience, music liking, music-trained individuals

## Abstract

**Introduction:**

Musical aesthetic emotions often display pleasant characteristics and function as a key reward mechanism in music appreciation. Previous research suggests that temporary mindfulness meditation training can enhance the aesthetic experiences associated with music aesthetic emotion processing (MAEP), fostering richer and more pleasant emotional responses. However, empirical evidence on the relationship between MAEP and mindfulness—particularly with respect to music genre, music training, and newly identified MAEP dimensions—remains limited. Therefore, this study, based on music-trained individuals and Chinese pop music, aims to investigate the effects of a 10-min mindfulness meditation training on MAEP.

**Methods:**

A 2 (group: Mindfulness Music Group [MMG] vs. Music Listening Group [MLG]) × 3 (music style: R&B/Pop/Folk) mixed experimental design was adopted. Sixty-three Chinese college students with professional music training were randomly assigned to MMG (Male = 15, Female = 16) or MLG (Male = 12, Female = 20). Psychological measures included the Geneva Emotional Music Scale (GEMS), the Five Facet Mindfulness Questionnaire (FFMQ), and the Positive and Negative Affect Schedule (PANAS). Data were analyzed using repeated measures analysis of variance and independent samples *t*-tests.

**Results:**

Self-report results indicated no significant between-group differences in PANAS or FFMQ scores (all *p* > 0.05). Aesthetic attitudes were found to be significantly positively correlated with emotional experiences, suggesting that aesthetic attitudes may represent a novel MAEP dimension. Additionally, temporary mindfulness meditation training significantly improved both aesthetic attitudes and aesthetic judgment scores across all three music styles. However, enhancements in emotional experiences were observed only for Chinese Folk music. Compared with the MLG, the MMG demonstrated greater attention and altered time perception, along with reduced bodily control during music listening.

**Conclusion:**

These findings provide new insights into the relationship between mindfulness and MAEP and highlight a potential new dimension—aesthetic attitudes—that may contribute to the psychological mechanisms underlying MAEP.

## 1 Introduction

Music listening is a pervasive and recurrent auditory activity in human society. This phenomenon can be understood through the concept of pleasure, defined as a subjective state that implies the associated behavior is rewarding and likely to be repeated (Salimpoor et al., 2009). Previous research has identified pleasant emotions as a crucial component of music enjoyment (Zatorre and Salimpoor, 2013). Emotional responses to artistic forms, such as films and plays, arise because the art diminishes the perception of its non-reality, immersing individuals in fictional contexts and enabling them to experience specific emotions (Frijda, 1989; Schindler et al., 2017; Menninghaus et al., 2019). Functional neuroimaging studies have further demonstrated that listening to music modulates activity in brain regions central to emotional processing, including the amygdala, nucleus accumbens, and hypothalamus (Koelsch, 2014). Music can induce both basic and complex emotions through various psychological mechanisms (Juslin and Västfjäll, 2008; Swaminathan and Schellenberg, 2015); however, it more frequently elicits aesthetic emotions rather than basic, discrete emotions (Scherer et al., 2001a; Scherer et al., 2001b). Notably, Menninghaus et al. (2019) provided a comprehensive and integrated discussion of aesthetic emotions and constructed a multidimensional model. Aesthetic emotions are emotions arising from an individual’s perception and evaluation of the intrinsic aesthetic appeal or virtues of a stimulus (Schindler et al., 2017; Scherer, 2005; Menninghaus et al., 2019). This characteristic distinguishes aesthetic emotions from utilitarian emotions, which emphasize the practical outcomes of objects in relation to personal goals. Aesthetic emotions focus on the object itself, whereas utilitarian emotions concern the consequences of the object (Schindler et al., 2017). These distinctions are supported by evidence suggesting that separate neural systems underlie aesthetic and utilitarian emotional processing (Chatterjee and Vartanian, 2014). Interestingly, unlike the negative bias found in classical emotion catalogues, aesthetic evaluation terminology contains far more positive emotions than negative ones. However, many overall positive aesthetic emotions also incorporate negative or mixed components (Menninghaus et al., 2019). Aesthetic emotions represent genuine emotional experiences, not merely perceived emotions. Gabrielsson (2001) argues that in music, emotion perception is a perceptual-cognitive process, while emotion experience is the emotional response evoked by music. General emotion models tend to emphasize negative emotions, limiting their capacity to explain widespread positive aesthetic emotions. Domain-specific models offer a more effective framework for understanding music-induced emotions (Zentner et al., 2008; Schindler et al., 2017; Menninghaus et al., 2019; Cowen et al., 2020) than basic emotion models (Ekman, 1992; Izard, 1977; Scherer et al., 2001a; Tracy and Randles, 2011; Cowen and Keltner, 2017) or dimensional emotion models (Russell, 2003).

Existing research indicates that musical aesthetic emotional processing occurs across multiple measurement dimensions at both psychological and behavioral levels (Nieminen et al., 2011, 2012; Menninghaus et al., 2019; Liu et al., 2021a; Liu et al., 2021b). Emotional experience, aesthetic judgment, and music liking are relatively common subjective experience dimensions studied in this context (Nieminen et al., 2011, 2012; Menninghaus et al., 2019; Liu et al., 2021a; Liu et al., 2021b). Juslin and Sloboda (2010) argue that aesthetic judgments, emotions, and musical preferences are partially independent phenomena but may interact through various mechanisms (pp. 605–642).

Emotional experience measures the emotions that participants actually feel in response to music. Previous studies have shown that emotions reported in self-report measures are often confused with perceived emotions (Gabrielsson, 2001; Scherer and Zentner, 2001; Schubert, 2013), while felt emotions are a stronger predictor of enjoyment (Schubert, 2007). Therefore, distinguishing between the felt and perceived emotions during music listening is an important control factor for ensuring the reliability and validity of emotional experience measurements (Scherer and Zentner, 2001). Additionally, music-induced emotions exhibit domain specificity, and everyday basic emotional terms (e.g., sadness, happiness, anger, or fear) inadequately capture the unique emotional qualities evoked by music (Juslin, 2013; Menninghaus et al., 2019; Scherer, 2004; Scherer and Zentner, 2001, 2008; Zentner et al., 2008). Consequently, the assessment of subjective experiences of musical aesthetic emotions requires domain-specific measurement tools (Scherer and Zentner, 2001; Zentner et al., 2008). Notably, Zentner et al. (2008) developed the Geneva Emotional Music Scale (GEMS), a tool specifically designed to measure musical emotions. Based on aesthetic emotion theory, GEMS involves listening to various music genres (Classical, Rock/Pop, Jazz, etc.), cluster analysis, and behavioral ratings of 425 emotion words, ultimately identifying essentially aesthetic—rather than utilitarian—musical emotions (wonder, transcendence, tenderness, nostalgia, peacefulness, power, joy, tension, and sadness), which goes beyond the emotions typically captured by general emotion models (Zentner et al., 2008; Juslin and Sloboda, 2010).

Aesthetic judgments of music are multidimensional, involving various subjective criteria related to aesthetic value (beauty, novelty, expression, emotion, style, technique, etc.) and individual differences in the relative weighting of these aesthetic standards (Juslin, 2013; Egermann and Reuben, 2020). Although research has identified beauty as the most central component of the concept of aesthetic value in music (Istók et al., 2009), this weighting may be relative (Juslin, 2013). Accordingly, aesthetic judgment can be defined as a value assessment based on an individual’s multidimensional aesthetic standards. Juslin (2013) further proposed a mechanism for music emotion induction—typically involving aesthetic emotions—mediated by aesthetic judgments. However, he also argued that aesthetic judgments do not necessarily induce emotions; emotions are triggered only when evaluation standards reach a certain threshold. This aesthetic emotion induction mechanism has been empirically supported in a limited number of studies (Egermann and Reuben, 2020). Musical preference reflects a conscious liking or disliking toward certain stimuli, is typically associated with the subjective experience of positive aesthetic emotions (Nieminen et al., 2011; Menninghaus et al., 2019; Egermann and Reuben, 2020), and reflects the approach or avoidance response to basic emotions (Swaminathan and Schellenberg, 2015). In music aesthetic emotional processing (MAEP) research, evidence regarding musical aesthetic attitudes remains sparse. Earlier work demonstrated that participants’ autonomous responses depended on their attitudes toward music (Harrer and Harrer, 1977). Furthermore, Juslin (2013) argued that aesthetic attitudes can enhance music perception and cognitive analysis, thereby informing aesthetic judgments. However, the emergence of aesthetic attitudes during music listening is not inevitable and appears to depend on specific aesthetic frameworks and perceptual salience features (e.g., novelty or extraordinary beauty) (Juslin, 2013). Thus, aesthetic attitudes may serve as a prerequisite for listeners to fully engage in musical aesthetic experiences.

Self-report remains the most effective and currently the sole available method for measuring individuals’ subjective emotional experience (Scherer and Zentner, 2001; Scherer, 2005). However, to ensure the reliability and validity of research findings, experimental control is essential when using this method (Scherer and Zentner, 2001; Scherer, 2005). Previous studies have found that MAEP is influenced by individual factors such as music selection (Blood and Zatorre, 2001; Juslin and Isaksson, 2014), familiarity (North and Hargreaves, 1995; Pereira et al., 2011), demand characteristics, the ability to distinguish between perceived and felt emotions (Gabrielsson, 2001; Scherer and Zentner, 2001; Schubert, 2013), mood state (Leder et al., 2004), and personality traits (Barrett et al., 2010). Objectively, a relatively safe listening environment is also a salient characteristic of aesthetic experiences.

Although Chinese popular music originated in the West, since the 21st century it has increasingly integrated a wide range of traditional Chinese cultural elements, thereby acquiring distinct cultural specificity (Lee and Wong, 2017; Lin, 2020; Yan, 2022; Tong and Ji, 2024; Luo et al., 2025). Menninghaus et al. (2019) suggested that cultural differences may have potential effects on aesthetic emotions. At present, research on MAEP has already produced numerous exceptional findings based on Western culture (Iwanaga et al., 2005; Konecni et al., 2007; Scherer and Zentner, 2008; Hunter et al., 2010; Trost et al., 2012). Within MAEP research, Chinese popular music remains an underexplored experimental stimulus. Although there is a well-established theoretical framework for addressing the conceptualization, induction mechanisms, classification, and measurement of music aesthetic emotions (Zentner et al., 2008; Meyer, 2008; Juslin, 2013; Coutinho and Scherer, 2017; Menninghaus et al., 2019), and numerous empirical studies have focused on music aesthetic experiences themselves, identifying causal relationships or correlations within MAEP (Nieminen et al., 2011; Egermann and Reuben, 2020), the potential influence of mindfulness meditation on the processing of musical aesthetic emotions remains underexplored (Liu et al., 2021a; Liu et al., 2021b). Based on the above considerations, the effects of temporary mindfulness meditation and Chinese popular music on MAEP may provide novel and cross-cultural evidence for understanding the psychological mechanisms underlying MAEP.

Mindfulness refers to a particular quality of attention and consciousness characterized by purposeful focus on the present moment and non-judgmental observation of experiences as they unfold. Mindfulness can be cultivated through various meditation practices (Baer, 2003; Kabat-Zinn, 2003). Operationally, mindfulness has been conceptualized in diverse ways: as a trait measured by instruments such as the Mindful Attention Awareness Scale, as a transient state following short-term mindfulness training, or as an intervention method (Davidson, 2010). Mindfulness meditation has yielded robust results in clinical interventions, emotion regulation, attention, and other areas (Brown and Ryan, 2003; Jha et al., 2007; Sahdra et al., 2011; Singh et al., 2014; Rodríguez-Carvajal, 2014; Knoerl et al., 2022). Previous studies have demonstrated that mindfulness meditation enhances emotional processing (Erisman and Roemer, 2010; Davidson, 2010; Tan et al., 2014; Bueno et al., 2015). Regarding the effective integration of music and mindfulness, Wolf (2019) proposed twelve methods—termed the “twelve bridges between music and mindfulness”—to connect these domains effectively. Recent research applying music listening across clinical, laboratory, and daily contexts has shown promising effects on emotion regulation and treatment of emotional disorders (Cooke et al., 2005; Nilsson, 2008; Belland et al., 2017). Furthermore, Eckhardt and Dinsmore (2012) proposed mindful music listening, combining mindfulness with music listening as a joint intervention for mental health, and achieved certain efficacy in alleviating depression. Studies examining the interactive effects of mindfulness meditation and specific music activities have shown that, compared to passive music listening, mindful music listening is more effective in promoting relaxation among typical adults (Goldberg, 2016), improving emotional regulation in elderly blind women (Chan et al., 2023), and alleviating chronic pain and anxiety (Young et al., 2025).

Mindfulness meditation training can be classified into three categories based on duration (Davidson, 2010; Davidson and Kaszniak, 2015; Sayers et al., 2015): temporary (3 min to 1 h), short-term (4 days to 4 months), and long-term (over 10 years). Evidence suggests that short-term mindfulness meditation training can influence emotional processing (Fredrickson et al., 2008; Basso, 2019). For example, short-term meditators, compared to control participants, exhibited significantly increased activation in the left anterior brain region, a pattern associated with enhanced positive emotions (Davidson et al., 2003). Temporary mindfulness meditation intervention has also been shown to affect emotional responses. In one study, it increased positive emotions during positive-emotion movie clips and decreased negative emotions during mixed-emotion movie clips (Erisman and Roemer, 2010). A functional magnetic resonance imaging (fMRI) study indicated that temporary mindfulness meditation intervention reduced activation in regions associated with emotional processing, such as the amygdala and parahippocampal gyrus (Lutz et al., 2014). These findings suggest that temporary mindfulness meditation is an effective strategy for modulating emotional processing (Broderick, 2005; Arch and Craske, 2006; Erisman and Roemer, 2010; Lutz et al., 2014).

Although these studies have demonstrated that mindfulness meditation training influences emotional processing (Broderick, 2005; Arch and Craske, 2006; Erisman and Roemer, 2010; Lutz et al., 2014) in both musically untrained individuals and in response to traditional Chinese folk instrumental pieces (Liu et al., 2021a; Liu et al., 2021b), little is known about its effects on MAEP (Liu et al., 2021a; Liu et al., 2021b). Furthermore, the relationship between mindfulness meditation and MAEP in Chinese popular music has not been explored. Therefore, this study aims to investigate the effects of temporary mindfulness meditation training on the MAEP of Chinese popular music in music-trained individuals, which may provide novel and cross-cultural evidence for understanding the psychological mechanisms underlying MAEP. The MAEP in this study includes four dimensions: aesthetic attitude, aesthetic judgment, emotional experience, and musical preference. Emotional experience is assessed using GEMS, a validated tool for measuring musical aesthetic emotions (Zentner et al., 2008). The other three dimensions are measured using Likert scales. The study will investigate differences in MAEP between the mindfulness music group (MMG) and music listening group (MLG) to evaluate the effects of temporary mindfulness meditation training on MAEP, attention, body control, and time perception in music-trained participants. Based on previous research, we hypothesize the following:

1. Compared to the MLG, the MMG will show significantly higher scores in aesthetic attitude, aesthetic judgment, and emotional experience, with no significant difference in music liking scores.
2. Compared to the MLG, the MMG will demonstrate significantly higher levels of attention and faster time perception, but significantly lower bodily control.
3. Aesthetic attitude will be significantly positively correlated with emotional experience, and aesthetic judgment will be significantly positively correlated with emotional experience.

## 2 Methods

### 2.1 Stimuli

#### 2.1.1 Mindful meditation audio

The type of meditation used in our study is a body scan meditation. The mindful meditation script was adapted from the English version of the mindful meditation script (Diaz, 2010). The script was translated into Chinese by two professional translators, then proofread and revised for accuracy and clarity. The mindful meditation audio was recorded in a professional recording studio by an experienced meditation and yoga instructor. Two recordings were produced: (1) a 10-min mindfulness meditation training audio, and (2) a 2-min mindfulness state maintenance audio. All recordings were saved in WAV format (sampling rate: 44.1 Hz, 16-bit, stereo, PCM encoding).

#### 2.1.2 Musical stimuli

**Figure 1:**
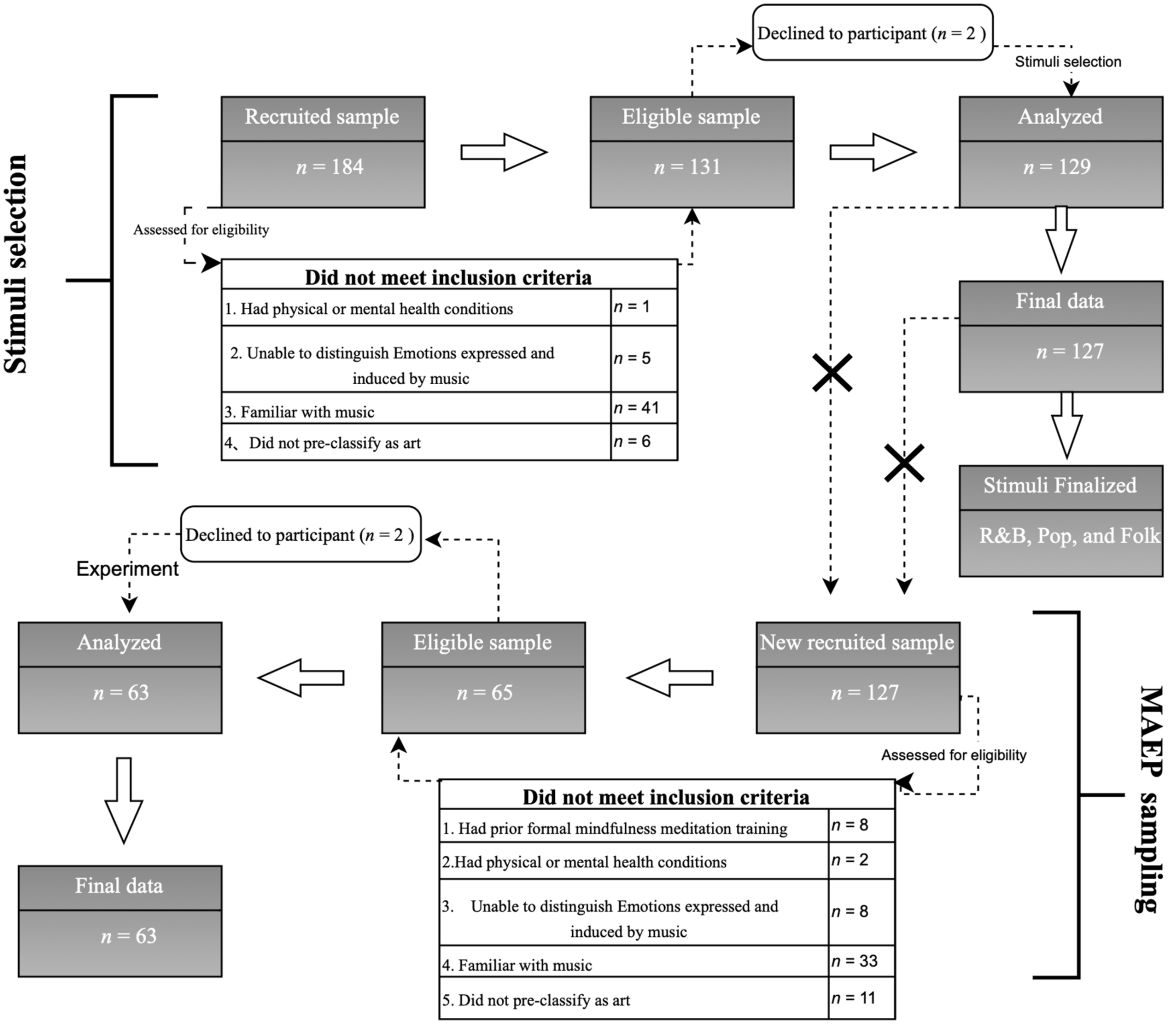
Sampling procedure for music material selection and experimental phase.

To ensure that the selected musical materials could effectively evoke musical aesthetic emotions, we recruited 184 undergraduate students from the Music College of Sichuan Normal University. After the eligibility screening (Figure 1), 131 participants met the inclusion criteria, and two declined to participate, resulting in a final sample of 129 participants. They were asked to rate six genres of Chinese popular music (R&B, Jazz, Rock, Folk, Blues, and Pop) in terms of aesthetic emotion induction. Ratings were made on a 6-point Likert scale ranging from 1 (very ineffective) to 6 (very effective). The primary research question was: “To what extent do the following six Chinese popular music genres evoke pleasant emotions during music listening?” The final valid dataset consisted of 127 participants who provided complete and valid data for analysis. A paired-sample *t*-test was conducted to compare the intensity of music aesthetic emotion induction among genres. Results indicated that R&B (*M* = 4.83, *SD* = 1.05) was rated significantly higher than Jazz (*M* = 4.51, *SD* = 1.21), *t*(126) = 2.78, *p* < 0.01; Rock (*M* = 4.38, *SD* = 1.36), *t*(126) = 3.42, *p* < 0.01; and Blues (*M* = 4.52, *SD* = 1.29), *t*(126) = 2.03, *p* < 0.05. However, R&B was rated significantly lower than Pop (*M* = 5.15, *SD* = 1.03), *t*(126) = 3.55, *p* < 0.01. Folk (*M* = 4.78, *SD* = 1.09) was rated significantly higher than Jazz, *t*(126) = 2.21, *p* < 0.05; Rock (*M* = 4.38, *SD* = 1.36), *t*(126) = 3.22, *p* < 0.01; and Blues (*M* = 4.52, *SD* = 1.29), *t*(126) = 3.22, *p* < 0.05, but significantly lower than Pop (*M* = 5.15, *SD* = 1.03), *t*(126) = 3.55, *p* < 0.01. No significant difference was found between Folk and R&B, *t*(126) = 0.40, *p* > 0.05. These findings indicate that in terms of music aesthetic emotion induction effectiveness: Pop > R&B ≈ Folk > Jazz, Rock, and Blues. Based on these results, we set Pop, R&B, and Folk as the three levels of the independent variable “music style,” with each level containing three one-minute music segments, totalling nine segments. In this study, “Pop” refers not to the broad category encompassing multiple substyles but to a specific form of contemporary Chinese popular music closely associated with professional pop performance and singing. All music excerpts were sourced from the QQ Music platform (Tencent Music Entertainment Co., Ltd.) and were representative of their respective styles. The original audio file was in FLAC format (a lossless audio compression format). To ensure high fluency and playback quality in E-prime 3.0, the FLAC format was converted to WAV format (sampling rate 44.1 Hz, 16-bit, stereo, PCM encoding format). To ensure high ecological validity, we excluded pieces strongly associated with specific contextual memories, thereby minimizing interference from music-related associations (Salimpoor et al., 2009). All participants confirmed that they were unfamiliar with all selected music excerpts.

### 2.2 Participants

The psychological mechanisms underlying MAEP in Chinese college students with musical training in Chinese popular music remain unclear, particularly regarding the potential influence of mindfulness meditation during music listening. Evidence from behavioral performance in this context is limited. To address this gap, this study recruited undergraduate students from the Music College of Sichuan Normal University as experimental participants and employed a mixed experimental design (Group = between; musical style = within). The required sample size for the repeated-measures design was calculated using G*Power 3.1. Based on an effect size of *f* = 0.25, *α* = 0.05, and Power (*1–β* err prob) = 0.80, with two groups and three measurements, corr among rep measures = 0.40, and *ε* = 0.90, the estimated total sample size required was *n* = 36. The inclusion criteria were as follows: (1) No prior formal mindfulness meditation training; (2) No physical or mental health conditions; (3) Ability to distinguish between emotions expressed by music and emotions induced by music (Scherer and Zentner, 2001); (4) No prior familiarity with the experimental music materials, including style, composer, performer, or title. A pre-experimental music familiarity survey was conducted to avoid the potential confounding effect of prior familiarity with the music on MAEP, as participants were music majors; (5) Belief that all experimental music stimuli belonged to the category of art. This final criterion (pre-classification) was adopted to ensure that aesthetic experiences could effectively emerge during the formal experiment (Leder et al., 2004; Juslin, 2013). To ensure the reliability and validity of the experimental results, participants who took part in the music material selection phase were excluded from the experimental phase. We recruited 127 undergraduate students from a music college, primarily due to methodological considerations. First, the musical familiarity (inclusion criteria 4), which was a controlled variable, might be relatively strict for individuals with musical training. Second, there could also be situations involving sample attrition and/or invalid data. Third, *f* used in G*Power was not based on a pilot study but on previous research; therefore, it might be overestimated in different contexts. Increasing the sample size helps compensate for this potential overestimation and maintain adequate statistical power. Therefore, we recruited 127 undergraduate students. After eligibility screening, 65 participants met the inclusion criteria, and 2 declined to participate. Sixty-three participants (*M* = 19.44 years, *SD* = 1.16; 36 females) were randomly assigned to either the experimental group (MMG; *n* = 31, 16 females) or the control group (MLG; *n* = 32, 20 females). The final valid dataset consisted of 63 participants (Figure 1). Prior to the formal experiment, an independent samples t-test was conducted to examine differences in music training duration (defined as the self-reported number of years of formal music training) between groups. No statistically significant difference in music training duration was observed between the experimental group (*M* = 5.53 years, *SD* = 2.29) and the control group (*M* = 5.03 years, *SD* = 2.39), *t*(61) = 0.85, *p* = 0.40, *d* = 0.21. All participants were right-handed, avoided substances or medications that might affect attention before the experiment, provided informed consent, and received compensation upon completing the experiment. This study was approved by the Sichuan Normal University Ethics Committee (IRB No. 2025LS0049).

### 2.3 MAEP tasks and measures

Based on the multi-component model of aesthetic emotions (Menninghaus et al., 2019) and the MAEP task developed in previous studies (Liu et al., 2021a; Liu et al., 2021b), a modified MAEP task was designed. This task includes four dimensions: aesthetic attitude, aesthetic judgment, emotional experience, and music liking. Aesthetic attitude refers to the degree of seriousness with which participants engage in the music listening process and is considered a prerequisite for the content input of aesthetic judgment (Juslin, 2013). Aesthetic judgment involves evaluating music based on diverse criteria (Juslin, 2013; Egermann and Reuben, 2020), and a series of criteria seem to be important in aesthetic judgment (Juslin and Isaksson, 2014). To avoid restricting assessments to the criterion of “beauty,” aesthetic judgment was measured on a 6-point Likert scale ranging from 1 (very unpleasant) to 6 (very pleasant). Emotional experience is viewed as the emotions experienced subjectively, driven by aesthetic judgment. Musical preference is considered a measure of conscious liking or disliking of certain stimuli, typically associated with a positive aesthetic emotion (Zentner et al., 2008). Aesthetic attitudes are rated on a 1–6 Likert scale, ranging from 1 (very careless) to 6 (very serious). Emotional experiences encompass nine dimensions, derived from the GEMS framework (Zentner et al., 2008), covering low arousal (peacefulness, nostalgia, sadness), medium arousal (wonder, transcendence, tenderness), and high arousal (tension, power, joy). Each emotion was rated on a 5-point scale ranging from 1 (not at all) to 5 (very much). Music liking were rated on a scale of 1 (very dislike) to 6 (very like). All dimensions were assessed *post hoc* to maintain ecological validity, overall coherence, and fluency in the music listening experience.

Both the experimental and control groups completed identical MAEP tasks and measurements, differing only in the instructional interface during the music listening session. Following a 10-min meditation training, the experimental group received the following instruction: “Next, please maintain the mindful state induced by the meditation training while completing the music listening task.” The control group received the instruction: “Please complete the music listening task.” After each music style listening session, both groups completed the MAEP post-assessment. For example, participants were instructed: “Please complete MAEP Post-Assessment 1. After completing ‘Assessment 1’, press the Q key to continue the experiment.”

### 2.4 Procedure

To minimize demand characteristics, we employed a double-blind experimental design (Scherer and Zentner, 2001). All participants were tested individually. Upon arrival, participants provided informed consent and completed the Positive and Negative Affect Schedule (PANAS). They were then seated in a comfortable recliner in the laboratory (Table 1). Prior to the experiment beginning, the laboratory temperature, headphone volume, and computer screen brightness were adjusted to individual comfort levels, with ambient noise levels maintained at 30–40 dB. Participants were allowed to adjust the headphone volume at any time during the experiment. The experiment was implemented using E-Prime 3.0, following a structured, standardized, and streamlined procedure. Both groups of participants were exposed to the musical styles in a fixed order (R&B → Pop → Folk) to ensure high levels of internal consistency and validity, facilitate precise replication, and avoid potential confounds that might arise from counterbalanced presentation. The experimental group received a 10-min mindfulness induction before the first style and a 2-min mindfulness state maintenance practice before each of the subsequent styles. After listening to each music style, participants completed the MAEP assessment (e.g., “Please complete MAEP Post-Assessment 1. After finishing ‘Assessment 1’, press the Q key to continue the experiment.”). Following the music listening tasks, participants in both groups were required to complete both the post-test for musical flow experience and the Five Facet Mindfulness Questionnaire. The post-test of musical flow experience assessed scores for attention level (1 = very low, 9 = very high), time perception (1 = very slow, 9 = very fast), and bodily awareness (1 = very relaxed, 9 = very tense). The corresponding prompts were: (1) “During the previous music listening process, what was your level of attention?”; (2) “During the music listening session you just completed, how much control did you have over your body?”; (3) “During the music listening session you just completed, how would you rate your perception of ‘time passing’?”

**Table 1:** Schematic of experimental procedure.

Phase	Step	MMG (*n* = 31) activity	MLG (*n* = 32) activity	Duration
1. Preparation	1	Signed informed consent and completed PANAS (Pre-test)	Signed informed consent and completed PANAS (Pre-test)	Self-paced
	2	Random assignment to groups	Random assignment to groups	-
2. Task module 1 (R&B)	3	10-min Mindfulness Induction	10-min Quiet Rest	10 min
	4	Listen to R&B music	Listen to R&B music	3 min
	5	MAEP Post-Assessment 1	MAEP Post-Assessment 1	Self-paced
3. Task module 2 (Pop)	6	2-min Mindfulness Maintenance	2-min Quiet Rest	2 min
	7	Listen to Pop music	Listen to Pop music	3 min
	8	MAEP Post-Assessment 2	MAEP Post-Assessment 2	Self-paced
4. Task module 3 (Folk)	9	2-min Mindfulness Maintenance	2-min Quiet Rest	2 min
	10	Listen to Folk music	Listen to Folk music	3 min
	11	MAEP Post-Assessment 3	MAEP Post-Assessment 3	Self-paced
5. Conclusion	12	Completed FFMQ and Musical Flow (Post-test)	Completed FFMQ and Musical Flow (Post-test)	Self-paced
	13	Debriefing and compensation	Debriefing and compensation	-

MMG, Mindfulness Music Group; MLG, Music Listening Group; PANAS, Positive and Negative Affect Schedule; MAEP, Musical Aesthetic Emotion Processing; FFMQ, Five Facet Mindfulness Questionnaire. “Self-paced” indicates the task duration was determined by the participant. “-” indicates duration was not applicable.

### 2.5 Self-report measures

#### 2.5.1 Five facet mindfulness questionnaire

The FFMQ is widely used to assess individual differences in mindfulness traits (Baer et al., 2006; Deng et al., 2011). The FFMQ was developed by integrating items from five existing mindfulness measures: the Mindful Attention Awareness Scale (MAAS), the Freiburg Mindfulness Inventory (FMI), the Kentucky Inventory of Mindfulness Skills (KIMS), the Cognitive and Affective Mindfulness Scale (CAMS), and the Mindfulness Questionnaire. Through exploratory and confirmatory factor analyses of these measurement tools, Baer et al. (2006) developed the 39-item FFMQ, which includes five measurement dimensions: (1) observing; (2) describing; (3) acting with awareness; (4) non-judging of inner experience; and (5) non-reactivity to inner experience. All items are rated on a five-point Likert scale ranging from 1 (never) to 5 (often), and the instrument demonstrates strong psychometric properties. Deng et al. (2011) adapted the FFMQ for use in Chinese populations, confirming its acceptable psychometric characteristics, as well as its high reliability and validity.

#### 2.5.2 Positive and negative affect schedule

Since an individual’s current emotional state may influence MAEP (Leder et al., 2004), participants’ emotional states will be assessed using PANAS prior to the experiment. PANAS is a reliable and valid measure of current affective states, consisting of two subscales: the Positive Affect Scale and the Negative Affect Scale, each consisting of 10 items (Watson et al., 1988). Participants rate each of the 20 adjectives on a scale from 1 (very slightly or not at all) to 5 (extremely).

### 2.6 Study design and data analysis

**Figure 2:**
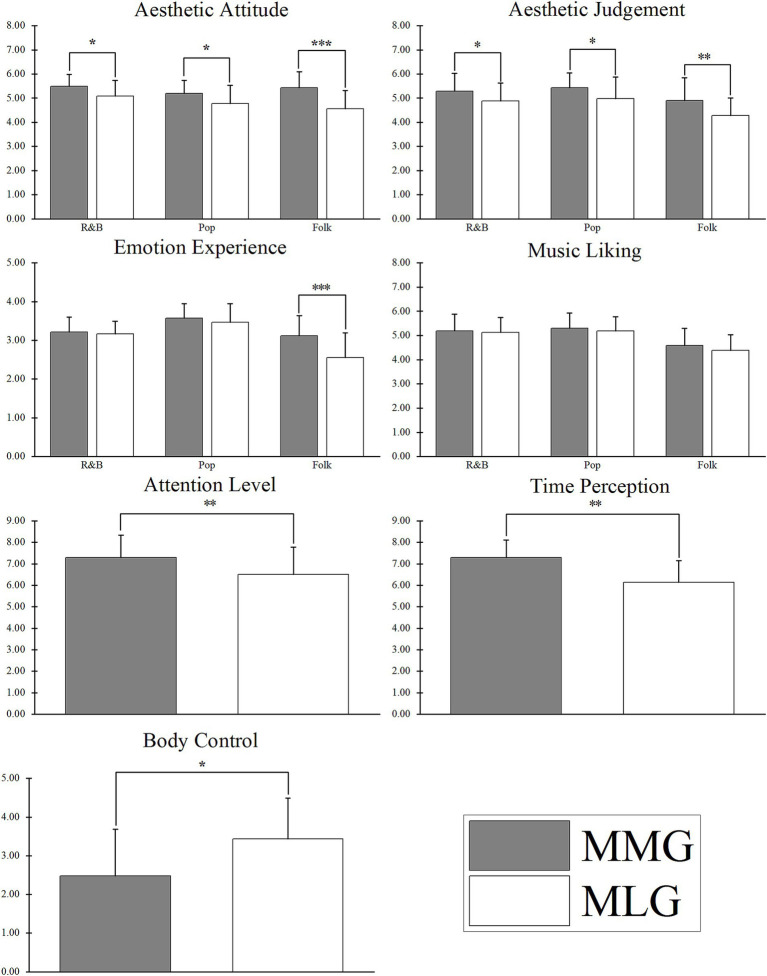
Differences between MMG and MLG in MAEP tasks with three music styles, attention level, time perception, and body control. Error bars represent mean ± SD; MMG, mindfulness music group; MLG, music listening group; ^*^p < 0.05, ^**^p < 0.01, ^***^p < 0.001.

This study employed a 2 (group: MMG vs. MLG) × 3 (music style: R&B/Pop/Folk) mixed experimental design. Participants were 63 Chinese college students with professional music training, randomly assigned to either the MMG (*n* = 31; 15 males, 16 females) or the MLG (*n* = 32; 12 males, 20 females). Data were analyzed using SPSS 22.0. All statistical assumptions were verified. Data met normality (Shapiro–Wilk test) and homogeneity of variance (Levene’s test) for t-tests and ANOVAs, and sphericity was confirmed by Mauchly’s test. *Post hoc* multiple comparisons were conducted using Bonferroni correction. For Pearson correlations, variables were normally distributed (all *p* > 0.05). Independent-samples *t*-tests were conducted to compare groups on demographic characteristics, FFMQ scores, and PANAS scores (Table 2). Repeated-measures analysis of variance (ANOVA) examined the main and interaction effects of group and music style, with group as the between-subjects factor and music style as the within-subjects factor (Table 3 and Figure 2). Pearson correlation analysis was used to assess relationships among aesthetic attitude, aesthetic judgment, and emotional experience within MAEP.

**Table 2:** Demographic information and self-report results of the participants.

Variable		MMG(M ± SD)	MLG(M ± SD)	*t*	*Cohen’s d*
		*n* = 31	*n* = 32		
Age		20 (1.51)	20.2 (1.20)		
Sex		Male = 15,female = 16	Male = 12,female = 20		
MTD		5.53 (2.29)	5.03 (2.39)	0.85	
PANAS		PA:3.27 (0.57) NA:1.77 (0.69)	PA:3.00 (0.69) NA:1.53 (0.49)	PA:1.67 NA:1.56	
FFMQ	Sum	116.18 (9.56)	116.09 (10.14)	0.01	
	OB	3.05 (0.45)	3.17 (0.41)	−0.34	
	DS	2.98 (0.55)	3.04 (0.58)	−0.13	
	AWA	3.09 (0.50)	3.01(0.47)	0.20	
	NJ	2.90(0.33)	3.00(0.29)	−0.39	
	NR	2.89(0.38)	2.95(0.43)	−0.18	
AL^**^		7.29(1.04)	6.50(1.27)	2.70	0.68
BC^*^		2.48(1.21)	3.44(1.05)	−3.36	0.85
TP^**^		7.26(0.82)	6.13(1.01)	4.90	1.23

MMG, mindful music group; MLG, music listening group; M, mean value; SD, standard deviation; MTD, music training duration (years of formal training); PANAS, positive and negative affect schedule; FFMQ, Five Facet Mindfulness Questionnaire (OB, Observing; DS, Describing; AWA, Acting with Awareness; NJ, Non-Judging of inner experience; NR, Non-Reactive to inner experience); AL, attention level; BC, body control; TP, time perception; *p < 0.05, **p < 0.01.

**Table 3:** MAEP task results of MMG and MLG.

Independent sample *t*-test result									
Dependent variable	MMG(M ± SD)			MLG(M ± SD)			*t*		
	Music style			Music style					
	R&B	Pop	Folk	R&B	Pop	Folk	R&B	Pop	Folk
AA^*^	5.48 (0.51)	5.19 (0.54)	5.42 (0.67)	5.09 (0.64)	4.78 (0.75)	4.56 (0.76)	2.67^*^	2.50^*^	4.73^***^
AJ^*^	5.29 (0.74)	5.42 (0.62)	4.90 (0.94)	4.88 (0.75)	4.97 (0.90)	4.28 (0.73)	2.21^*^	2.32^*^	2.93^**^
EE	3.21 (0.39)	3.57 (0.38)	3.12 (0.52)	3.16 (0.33)	3.46 (0.49)	2.56 (0.63)	0.60	1.00	3.82^***^
ML	5.19 (0.70)	5.29 (0.64)	4.58 (0.72)	5.13 (0.61)	5.19 (0.59)	4.38 (0.66)	0.41	0.66	1.18

Results of repeated measures analysis of variance									
Dependent variable	Main effect of group			Main effect of music style			Interaction effect		
	*df*	*F*	$\eta_{p}^{2}$	*df*	*F*	$\eta_{p}^{2}$	*df*	*F*	$\eta_{p}^{2}$
AA^*^	(1.61)	24.81^***^	0.29	(2.61)	5.39^**^	0.08	(2.61)	3.12^*^	0.05
AJ	(1.61)	16.25^***^	0.21	(2.61)	11.26^***^	0.16	(2.61)	0.34	0.01
EE^**^	(1.61)	9.86^**^	0.14	(2.61)	37.66^***^	0.38	(2.61)	6.32^**^	0.09
ML	(1.61)	1.44	0.02	(2.61)	28.57^***^	0.32	(2.61)	0.21	0.003

MMG, mindful meditation group; MLG, music listening group; M, mean value; SD, standard deviation; AA, aesthetic attitude; AJ, aesthetic judgement; EE, emotion experience; ML, music liking; df, degree of freedom; ^*^p < 0.05, ^**^p < 0.01, ^***^p < 0.001.

## 3 Results

### 3.1 Self-reported results

The demographic information and self-report results of participants are shown in Table 2 and Figure 2. Independent samples *t*-tests yielded the following results: (1) No significant group differences were found in self-reported music training duration (*t* = −0.85, *df* = 61, *p* = 0.40, *d =* −0.21); (2) PANAS scores revealed no significant group differences in positive (*t* = 1.67, *df* = 61, *p* = 0.10, *d* = 0.43) or negative affect (*t* = 1.56, *df* = 54.44, *p* = 0.124, *d* = 0.40); (3) FFMQ scores showed no significant group differences across all subscales (all *p* > 0.05); (4) Post-test scores indicated that MMG participants exhibited significantly higher levels of attention during music listening compared to MLG participants (*t* = 2.70, *df* = 61, *p* = 0.009, *d* = 0.68). Regarding time perception and bodily control, MMG participants reported a faster perceived passage of musical time (*t* = −3.36, *df* = 61, *p* < 0.001, *d* = 0.85) and a more relaxed bodily state (*t* = 4.90, *df* = 61, *p* = 0.001, *d* = 1.23).

### 3.2 MAEP task results

The behavioral results of MAEP of the participants at the three music styles are shown in Table 3.

#### 3.2.1 Aesthetic attitude

A repeated-measures ANOVA revealed a significant main effect of group, *F*_(1,61)_ = 24.81, *p* < 0.001, $\eta_{p}^{2}$ = 0.25. *Post hoc t*-tests indicated that R&B (*t* = 2.67, *df* = 61, *p* = 0.01, *d* = 0.67), Pop (*t* = 2.50, *df* = 56.49, *p* < 0.05, *d* = 0.63), and Folk (*t* = 4.74, *df* = 61, *p* < 0.001, *d* = 1.20) aesthetic attitude scores were significantly higher in MMG than in MLG. The main effect of music style was also significant, *F*_(2,61)_ = 5.39, *p* < 0.01, $\eta_{p}^{2}$ = 0.08. Pairwise comparisons of within-subject factors showed that Pop (*p* = 0.22) and Folk (*p* = 0.21) were significantly lower than R&B in aesthetic attitude scores; however, there was no significant difference between Pop and Folk (*p* = 1.000). The interaction effect between group and music style was significant, *F*_(2,61)_ = 3.12, *p* = 0.045, $\eta_{p}^{2}$ = 0.05.

#### 3.2.2 Aesthetic judgment

Repeated-measures ANOVA showed a significant main effect of group, *F*_(1,61)_ = 16.25, *p* < 0.001, $\eta_{p}^{2}$ = 0.21. Post hoc *t*-tests revealed that the aesthetic judgment scores for all three music styles were significantly higher in MMG than in MLG: R&B (*t* = 2.21, *df* = 61, *p* = 0.031, *d* = 0.55), Pop (*t* = 2.32, *df* = 55.24, *p* = 0.024, *d* = 0.58), and Folk (*t* = 2.93, *df* = 61, *p* = 0.005, *d* = 0.74). A significant main effect of music style was also observed, *F*_(2,61)_ = 11.26, *p* < 0.001, $\eta_{p}^{2}$ = 0.16. Pairwise comparisons of within-subject factors showed that R&B (*p* = 0.002) and Pop (*p* < 0.001) scored significantly higher than Folk in aesthetic judgment scores, with no significant difference between R&B and Pop (*p* = 1.000). No significant interaction effects were found between groups and music style, *F*_(2,61)_ = 0.34, *p* = 0.716, $\eta_{p}^{2}$ = 0.01.

#### 3.2.3 Emotion experience

Repeated-measures ANOVA revealed significant main effects of group, with *F*_(1,61)_ = 9.86, *p* = 0.003, $\eta_{p}^{2}$ = 0.14. Post hoc *t*-tests indicated that MMG scored significantly higher than MLG on Folk emotional experience scores, *t*_(61)_ *=* 3.82, *p* < 0.001, *d* = 0.97, with no significant differences between the other two music styles (R&B, *p* = 0.552; Pop, *p* = 0.322). The main effect of music style was significant, F_(2,61)_ = 37.66, *p* < 0.001, $\eta_{p}^{2}$ = 0.38. Pairwise comparisons of within-subject factors showed that emotional experience scores decreased in the following order: Pop > R&B > Folk (all *p* < 0.001). The interaction effect between group and music style was significant, *F*_(2,61)_ = 6.32, *p* = 0.002, $\eta_{p}^{2}$ = 0.09.

#### 3.2.4 Relationship between aesthetic attitude and emotional experience

Pearson correlation analysis revealed that aesthetic attitude and emotion experience were significantly positively correlated for all music styles (R&B, *r* = 0.35, *p* = 0.005; Pop, *r* = 0.37, *p* = 0.004; and Folk, *r* = 0.57, *p* < 0.001).

#### 3.2.5 Relationship between aesthetic judgment and emotional experience

Pearson correlation analysis showed that aesthetic judgment was significantly positively correlated with emotional experience for all music styles (R&B, *r* = 0.59, *p* < 0.001; Pop, *r* = 0.43, *p* < 0.001; and Folk, *r* = 0.55, *p* < 0.001).

#### 3.2.6 Music liking

Repeated-measures ANOVA revealed no significant main effect of group, with *F*_(1,61)_ = 1.44, *p* = 0.235, $\eta_{p}^{2}$ = 0.02. Post hoc *t*-tests indicated no significant group differences in music liking scores among the three music styles: R&B (*t =* 0.41, *df* = 61, *p* = 0.680, *d* = 0.11), Pop (*t =* 0.66, *df* = 61, *p* = 0.511, *d* = 0.16), and Folk (*t =* 1.18, *df* = 61, *p* = 0.242, *d* = 0.29). However, the main effect of music style was significant, *F*_(2,61)_ = 28.57, *p* < 0.001, $\eta_{p}^{2}$ = 0.32. Pairwise comparisons of within-subject factors indicated that R&B and Pop were significantly higher than Folk in music liking scores (all *p* < 0.001), with no significant difference in music liking scores between R&B and Pop (*p* = 1.000). The interaction effect between group and music style was not significant, *F*_(2, 61)_ = 0.21, *p* = 0.813, $\eta_{p}^{2}$ = 0.003.

## 4 Discussion

This study’s primary objective was to explore the potential effects of temporary mindfulness meditation on MAEP in individuals undergoing music training. Therefore, we compared the MMG and MLG in terms of differences in various dimensions of MAEP, attention levels, time perception, and body control. The results largely support our research hypotheses. Our findings indicate that: (1) Aesthetic attitudes, aesthetic judgments, and emotional experiences were influenced by temporary mindfulness meditation training. Under mindfulness conditions, aesthetic attitudes and judgments toward three types of Chinese popular music were significantly enhanced, and emotional experiences toward folk music were notably improved, while music liking showed no significant differences; (2) Temporary mindfulness meditation training significantly improved attention levels and time perception during music listening, but reduced body control; (3) Aesthetic attitudes were significantly positively correlated with emotional experiences, and aesthetic judgments were significantly positively correlated with emotional experiences.

Aesthetic attitudes may represent an important dimension of MAEP (Juslin, 2013). Our study confirmed the existence of this dimension and demonstrated a significant positive correlation between aesthetic attitudes and emotional experiences. This may be because the degree of aesthetic attitudes determines the richness of aesthetic perceptual and cognitive information input, thereby influencing the subjective emotional experiences driven by aesthetic judgments (Juslin, 2013). According to previous research, aesthetic attitudes may be preceded by a pre-classification process in which listeners determine whether a musical work is to be regarded as art (Leder et al., 2004; Juslin, 2013). Once categorized as art, aesthetic attitudes are more likely to emerge (Juslin, 2013). In this study, all participants categorized all experimental musical stimuli as art, thereby establishing the necessary precondition for the emergence of aesthetic attitudes. We observed significant main effects of group on this dimension, indicating that temporary mindfulness meditation training significantly enhanced listeners’ aesthetic attitudes. The mindful state is characterized by curiosity, openness, and acceptance toward present experiences (Bishop et al., 2004). We propose that the observed enhancement of aesthetic attitudes in this study may be attributed to these qualities of mindfulness, in combination with the pre-classification of the musical works as art.

Our research found that the aesthetic judgments of individuals with musical training were significantly enhanced following temporary mindfulness meditation. This finding aligns with previous studies (Liu et al., 2021a; Liu et al., 2021b), which demonstrated that temporary mindfulness meditation and musical emotion levels (happiness, peacefulness, sadness) influenced the MAEP of traditional Chinese folk instrumental works, and that the aesthetic experience of non-musically trained individuals was enhanced (Liu et al., 2021a; Liu et al., 2021b). Moreover, prior research has suggested that aesthetic judgments may serve as a key mechanism for inducing musical aesthetic emotions (Juslin, 2013; Egermann and Reuben, 2020), an aspect we also examined. In our study, aesthetic judgments were significantly and positively correlated with emotional experiences when listening to Chinese R&B, Pop, and Folk music. Notably, the aesthetic judgment scores in our study were relatively high, suggesting that participants’ aesthetic standards were well aligned, thereby enabling aesthetic judgments in music listening to reach a specific threshold for eliciting aesthetic emotions (Juslin, 2013). This scoring pattern was also observed in a related study (Liu et al., 2021a; Liu et al., 2021b). Emotional experiences, as defined in this context, refer to the actual emotions felt by individuals during music listening, rather than merely perceived emotions (Gabrielsson, 2001; Scherer and Zentner, 2001; Schubert, 2013; Schindler et al., 2017). Music-induced emotions demonstrate domain specificity, and common basic emotion terms (e.g., sadness, happiness, anger, or fear) are often insufficient to capture their unique qualities (Juslin, 2013; Menninghaus et al., 2019; Scherer, 2004; Scherer and Zentner, 2001, 2008; Zentner et al., 2008). Our hypothesis regarding emotional experiences was only partially supported. No significant group differences emerged for R&B and Pop, indicating that temporary mindfulness meditation training did not significantly enhance emotional experiences in these genres among musically trained individuals. However, emotional experiences during Folk listening were enhanced, with a significant group main effect in this dimension. This suggests that the emotional experiences of musically trained individuals can be significantly influenced by temporary mindfulness meditation, possibly due to heightened attentional engagement during listening. Furthermore, participants’ professional music training may have influenced the observed differences between R&B and Pop (Brattico et al., 2016). Previous studies have shown that music liking reflects one’s evaluative stance toward musical stimuli and is generally associated with the subjective experience of positive aesthetic emotions (Nieminen et al., 2011; Menninghaus et al., 2019; Egermann and Reuben, 2020), and also reflects basic approach–avoidance responses (Swaminathan and Schellenberg, 2015). Related work has also found significant positive effects of temporary mindfulness meditation on music liking (Liu et al., 2021a; Liu et al., 2021b). However, we observed no significant differences in music liking between groups. This discrepancy may be related to individual personality traits and their influence on music type preferences (Cattell and Anderson, 1953; Dollinger, 1993; Rentfrow and Gosling, 2003; Chamorro-Premuzic and Furnham, 2007; Greenberg et al., 2015).

Previous studies have shown that individuals can sustain a present-moment mind–body state, remain open to more positive experiences, and maintain an internal experiential state free from judgment (Davidson, 2010; Davidson and Kaszniak, 2015; Sayers et al., 2015). Our results indicate that, compared with the MLG, mindfulness meditation training significantly enhanced attention levels during the aesthetic experience process of the MMG. Participants in the MMG also perceived musical time as passing more quickly and reported lower levels of bodily control. The reduction in bodily control may reflect a conscious state of relaxation during music listening (Diaz, 2013; Rodríguez-Carvajal, 2014; Liu et al., 2021a; Liu et al., 2021b). Furthermore, previous research has shown that time perception is closely related to emotional states, which can alter the perception of musical time. The accelerated perception of musical time in the MMG may be attributed to moment-to-moment awareness during positive emotional experiences.

It is worth noting that our study did not include pre- and post-tests of state mindfulness between the two groups. This is a limitation of our study. However, post-tests of attentional level and bodily awareness showed that, compared with the MLG, the MMG experienced higher attentional levels and a more relaxed bodily state during the aesthetic experience. Although there were no direct indicators, attentional focus and bodily relaxation are core dimensions of mindfulness (Bishop et al., 2004; Davidson, 2010; Basso, 2019), which may serve as indirect feedback suggesting that the state of mindfulness was effectively improved. Some studies have found no evidence that temporary mindfulness meditation affects trait mindfulness; rather, it is more likely to enhance state mindfulness levels (Palmer et al., 2023; Sleimen-Malkoun et al., 2023; Somaraju et al., 2023). In addition, trait mindfulness may be a potential moderator variable (Palmer et al., 2023). The results showed no significant difference in trait mindfulness levels between the two groups, which to some extent reduced the influence of individual trait differences on the experimental outcomes. This finding is consistent with previous studies (Palmer et al., 2023; Sleimen-Malkoun et al., 2023; Somaraju et al., 2023). For methodological considerations, we measured trait mindfulness only in the post-test to avoid possible demand characteristics that could influence the experimental results. There was no significant difference in PANAS scores between the two groups, indicating successful random assignment. This, to some extent, minimized the potential confounding effects of positive and negative affect differences on the experimental results, thereby strengthening the likelihood that the observed variability between the two groups could be attributed to the brief mindfulness meditation intervention. In addition, our musical materials were presented in a fixed order. Although this approach ensured a high level of internal consistency in the “induction–maintenance” intervention procedure, it also introduced potential confounding effects of order and dosage that cannot be ruled out. For instance, in our experiment, Folk music was always presented last, corresponding to the longest mindfulness intervention dosage (14 min). We found that only in the Folk music condition did the MMG show significantly higher emotional experience scores than the MLG. Therefore, we acknowledge that this result may have been influenced by a fatigue effect (e.g., lower MLG scores due to fatigue) or a cumulative dosage effect (e.g., higher MMG scores due to the 14-min intervention), or possibly both.

## 5 Conclusion

This study is the first to demonstrate that temporary mindfulness meditation significantly enhances MAEP in listeners of Chinese popular music. The results indicate that a single 10-min temporary mindfulness meditation training effectively enhances aesthetic attitudes, aesthetic judgments, and emotional experiences toward Chinese pop music style (particularly Chinese folk music) among music-trained individuals, while also improving attention levels during listening (*d* = *0.*68), accelerating time perception (*d* = 1.23), and reducing body control (*d* = *0.*85), supporting the hypothesis that mindfulness deepens aesthetic experiences by optimizing cognitive and physiological states (Diaz, 2013; Liu et al., 2021a; Liu et al., 2021b). Furthermore, this study provides empirical evidence that aesthetic attitudes constitute a distinct dimension of MAEP and that aesthetic judgments are positively correlated with emotional experiences (*r* = 0.35–0.57), thereby supporting key theoretical propositions by Juslin (2013). The findings address a notable gap in cross-cultural research on aesthetic emotion in Chinese popular music (Zentner et al., 2008) and highlight the potential application of mindfulness meditation as a tool in both music education and clinical intervention. Research limitations include: (1) The sample was restricted to music majors from a single university. Future studies should include diverse age groups and non-professional populations; (2) Reliance on self-report methods may introduce subjective biases. Combining behavioral assessments with neuroimaging techniques such as fMRI or event-related potential could provide more objective insights into the neural mechanisms underlying emotion-related brain regions (e.g., amygdala and nucleus accumbens) (Koelsch, 2014); (3) The study examined only the effects of temporary mindfulness meditation on MAEP. Future research should compare short-term and long-term mindfulness meditation training; (4) Only indirect indicators were used as evidence, and a formal mindfulness state check was lacking. Future research should include a formal mindfulness manipulation check; (5) Using a fixed order to present experimental materials makes it difficult to avoid order or dosage confounds. Future studies could employ a counterbalanced presentation to provide more robust evidence. This brief 10-min, easy-to-implement intervention has direct application value for music education and clinical interventions. For example, mindfulness induction before music appreciation may help students quickly switch from daily thinking to an aesthetic mode. Similarly, temporary meditation training before music therapy could increase patients’ acceptance of therapeutic music, thereby enhancing the therapeutic effect. Cross-cultural comparisons across various music types and investigations into the long-term effects of mindfulness meditation on both musical aesthetic experiences and mental health represent promising directions for further research (Menninghaus et al., 2019).

## Data Availability

The original contributions presented in the study are included in the article/supplementary material, further inquiries can be directed to the corresponding author.

## References

[ref1] Arch J. J. Craske M. G. (2006). Mechanisms of mindfulness: emotion regulation following a focused breathing induction. Behav. Res. Ther. 44, 1849–1858. doi: 10.1016/j.brat.2005.12.007, 16460668

[ref9001] Baer R. A. (2003). Mindfulness training as a clinical intervention: A conceptual and empirical review. Clinical Psychology: Science and Practice, 10, 125–143. doi: 10.1093/clipsy.bpg015

[ref2] Baer R. A. Smith G. T. Hopkins J. Krietemeyer J. Toney L. (2006). Using self-report assess-ment methods to explore facets of mindfulness. Assessment 13, 27–45. doi: 10.1177/1073191105283504 16443717

[ref3] Barrett F. S. Grimm K. J. Robins R. W. Wildschut T. Sedikides C. Janata P. (2010). Music-evoked nostalgia: affect, memory, and personality. Emotion 10, 390–403. doi: 10.1037/a0019006, 20515227

[ref4] Basso J. C. (2019). Brief, daily meditation enhances attention, memory, mood, and emotional regulation in non-experienced meditators. Behav. Brain Res. 356, 208–220. doi: 10.1016/j.bbr.2018.08.023, 30153464

[ref5] Belland L. Rivera-Reyes L. Hwang U. (2017). Using music to reduce anxiety among older adults in the emergency department: a randomized pilot study. J. Integr. Med. 15, 450–455. doi: 10.1016/S2095-4964(17)60341-8, 29103414

[ref6] Bishop S. R. Lau M. Shapiro S. Carlson L. Anderson N. D. Carmody J. . (2004). Mindfulness: a proposed operational definition. Clin. Psychol. Sci. Pract. 11, 230–241. doi: 10.1093/clipsy.bph077, 41281248

[ref7] Blood A. J. Zatorre R. J. (2001). Intensely pleasurable responses to music correlate with activity in brain regions implicated in reward and emotion. Proc. Natl. Acad. Sci. 98, 11818–11823. doi: 10.1073/pnas.191355898, 11573015 PMC58814

[ref8] Brattico E. Bogert B. Alluri V. Tervaniemi M. Eerola T. Jacobsen T. (2016). It’s sad but I like it: the neural dissociation between musical emotions and liking in experts and laypersons. Front. Hum. Neurosci. 9:676. doi: 10.3389/fnhum.2015.00676, 26778996 PMC4701928

[ref9] Broderick P. C. (2005). Mindfulness and coping with dysphoric mood: contrasts with rumination and distraction. Cogn. Ther. Res. 29, 501–510. doi: 10.1007/s10608-005-3888-0

[ref10] Brown K. W. Ryan R. M. (2003). The benefits of being present: mindfulness and its role in psychological well-being. J. Pers. Soc. Psychol. 84, 822–848. doi: 10.1037/0022-3514.84.4.822, 12703651

[ref11] Bueno V. F. Kozasa E. H. Da Silva M. A. Alves T. M. Louzã M. R. Pompéia S. (2015). Mi-ndfulness meditation improves mood, quality of life, and attention in adults with attention deficit hyperactivity disorder. Biomed. Res. Int. 2015, 1–14. doi: 10.1155/2015/962857, 26137496 PMC4475526

[ref12] Cattell R. B. Anderson J. C. (1953). The measurement of personality and behavior disorders by the I. P. A. T. Music preference test. J. Appl. Psychol. 37, 446–454. doi: 10.1037/h0056224

[ref9003] Chamorro-Premuzic T. Furnham A. (2007). Personality and music: Can traits explain how people use music in everyday life? British Journal of Psychology, 98, 175–185. doi: 10.1348/000712606X111177 17456267

[ref13] Chan S. H. W. Cheung M. Y. C. Chiu A. T. S. Leung M. H. T. Kuo M. C. C. Yip D. Y. C. . (2023). Clinical effectiveness of mindfulness-based music therapy on improving emotional regulation in blind older women: a randomized controlled trial. Integr. Med. Res. 12:100993. doi: 10.1016/j.imr.2023.100993 37915438 PMC10616413

[ref14] Chatterjee A. Vartanian O. (2014). Neuroaesthetics. Trends Cogn. Sci. 18, 370–375. doi: 10.1016/j.tics.2014.03.003, 24768244

[ref15] Cooke M. Chaboyer W. Hiratos M. A. (2005). Music and its effect on anxiety in short waiting periods: a critical appraisal. J. Clin. Nurs. 14, 145–155. doi: 10.1111/j.1365-2702.2004.01033.x, 15669923

[ref16] Coutinho E. Scherer K. R. (2017). Introducing the GEneva music-induced affect checklist (GEMIAC). Music. Percept. 34, 371–386. doi: 10.1525/mp.2017.34.4.371

[ref17] Cowen A. S. Fang X. Sauter D. Keltner D. (2020). What music makes us feel: at least 13 di- mensions organize subjective experiences associated with music across different cultures. Proc. Natl. Acad. Sci. USA 117, 1924–1934. doi: 10.1073/pnas.1910704117, 31907316 PMC6995018

[ref9002] Cowen A. S. Keltner D. (2017). Self-report captures 27 distinct categories of emotion bridged by continuous gradients. Proceedings of the National Academy of Sciences, 114, 2017–2022. doi: 10.1073/pnas.1702247114 PMC5617253 28874542

[ref18] Davidson R. J. (2010). Empirical explorations of mindfulness: conceptual and methodological conundrums. Emotion 10, 8–11. doi: 10.1037/a0018480, 20141297 PMC4485421

[ref20] Davidson R. J. Kaszniak A. W. (2015). Conceptual and methodological issues in research on mi-ndfulness and meditation. Am. Psychol. 70, 581–592. doi: 10.1037/a0039512, 26436310 PMC4627495

[ref19] Davidson R. J. Kabat-Zinn J. Schumacher J. Rosenkranz M. Muller D. Santorelli S. F. . (2003). Alterations in brain and immune function produced by mindfulness meditation. Psychosom. Med. 65, 564–570. doi: 10.1097/01.psy.0000077505.67574.e3, 12883106

[ref21] Deng Y.-Q. Liu X.-H. Rodriguez M. A. Xia C.-Y. (2011). The five facet mindfulness questionnaire: psychometric properties of the Chinese version. Mindfulness 2, 123–128. doi: 10.1007/s12671-011-0050-9

[ref22] Diaz F. M. (2010) A preliminary investigation into the effects of a brief mindfulness induction on perceptions of attention, aesthetic response, and flow during music listening [doctoral dissertation, the Florida State University]. In ProQuest dissertations and theses. Available online at: https://www.proquest.com/docview/875951213/abstract/318CB38607444352PQ/1 (Accessed January 15, 2025).

[ref23] Diaz F. M. (2013). Mindfulness, attention, and flow during music listening: an empirical investigation. Psychol. Music 41, 42–58. doi: 10.1177/0305735611415144

[ref24] Dollinger S. J. (1993). Research note: personality and music preference: extraversion and excitement seeking or openness to experience? Psychol. Music 21, 73–77. doi: 10.1177/030573569302100105

[ref25] Eckhardt K. J. Dinsmore J. A. (2012). Mindful music listening as a potential treatment for depre-ssion. J. Creat. Ment. Health 7, 175–186. doi: 10.1080/15401383.2012.685020

[ref26] Egermann H. Reuben F. (2020). “Beauty is how you feel inside”: aesthetic judgments are related to emotional responses to contemporary music. Front. Psychol. 11:29. doi: 10.3389/fpsyg.2020.510029, 33281651 PMC7691637

[ref27] Ekman P. (1992). An argument for basic emotions. Cognit. Emot. 6, 169–200. doi: 10.1080/02699939208411068, 41277151

[ref28] Erisman S. M. Roemer L. (2010). A preliminary investigation of the effects of experimentally induced mindfulness on emotional responding to film clips. Emotion 10, 72–82. doi: 10.1037/a0017162, 20141304 PMC2868364

[ref29] Fredrickson B. L. Cohn M. A. Coffey K. A. Pek J. Finkel S. M. (2008). Open hearts build li-ves: positive emotions, induced through loving-kindness meditation, build consequential personal resources. J. Pers. Soc. Psychol. 95, 1045–1062. doi: 10.1037/a0013262, 18954193 PMC3156028

[ref30] Frijda N. H. (1989). Aesthetic emotions and reality. Am. Psychol. 44, 1546–1547. doi: 10.1037/0003-066X.44.12.1546

[ref31] Gabrielsson A. (2001). Emotion perceived and emotion felt: same or different? Musicae Sci. 5, 123–147. doi: 10.1177/10298649020050S105

[ref32] Goldberg A. R. (2016). Preferred music-based mindfulness: a new intervention for stress reduction [ProQuest Information & Learning]. In dissertation abstracts international: section B: the Scien-ces and engineering (Vol. 76, issues 11-B(E)). Available online at: https://www.proquest.com/docview/1798731482/AF977756E07C4F71PQ/1?sourcetype=Dissertations%20&%20Theses (Accessed January 20, 2025).

[ref9005] Greenberg D. M. Baron-Cohen S. Stillwell D. J. Kosinski M. Rentfrow P. J. (2015). Musical preferences are linked to cognitive styles. PLOS ONE, 10:e0131151. doi: 10.1371/journal.pone.0131151 26200656 PMC4511638

[ref33] Harrer G. Harrer H. (1977). “Music, emotion and autonomic function” in Music and the brain. eds. Critchley M. Henson R. A. (London: Elsevier), 202–216. doi: 10.1016/B978-0-433-06703-0.50019-X

[ref34] Hunter P. G. Schellenberg E. G. Schimmack U. (2010). Feelings and perceptions of happiness and sadness induced by music: similarities, differences, and mixed emotions. Psychol. Aesthet. Creat. Arts 4, 47–56. doi: 10.1037/a0016873

[ref35] Istók E. Brattico E. Jacobsen T. Krohn K. Müller M. Tervaniemi M. (2009). Aesthetic resp-onses to music: a questionnaire study. Music. Sci. 13, 183–206. doi: 10.1177/102986490901300201

[ref36] Iwanaga M. Kobayashi A. Kawasaki C. (2005). Heart rate variability with repetitive exposure to music. Biol. Psychol. 70, 61–66. doi: 10.1016/j.biopsycho.2004.11.015, 16038775

[ref37] Izard C. E. (1977). Human Emotions. New York: Springer US.

[ref38] Jha A. P. Krompinger J. Baime M. J. (2007). Mindfulness training modifies subsystems of atten-tion. Cogn. Affect. Behav. Neurosci. 7, 109–119. doi: 10.3758/CABN.7.2.109, 17672382

[ref39] Juslin P. N. (2013). From everyday emotions to aesthetic emotions: towards a unified theory of mus-ical emotions. Phys Life Rev 10, 235–266. doi: 10.1016/j.plrev.2013.05.008, 23769678

[ref40] Juslin P. N. Isaksson S. (2014). Subjective criteria for choice and aesthetic judgment of music: a comparison of psychology and music students. Res. Stud. Music Educ. 36, 179–198. doi: 10.1177/1321103x14540259

[ref41] Juslin P. N. Sloboda J. A. (2010). Handbook of music and emotion: Theory, research, and applications. Oxford: Oxford University Press.

[ref42] Juslin P. N. Västfjäll D. (2008). Emotional responses to music: the need to consider underlying mechanisms. Behav. Brain Sci. 31, 559–575. doi: 10.1017/S0140525X08005293, 18826699

[ref9006] Kabat-Zinn J. (2003). Mindfulness-based interventions in context: Past, present, and future. Clinical Psychology: Science and Practice, 10, 144–156. doi: 10.1093/clipsy.bpg016

[ref43] Knoerl R. Mazzola E. Woods H. Buchbinder E. Frazier L. LaCasce A. . (2022). Exploring the feasibility of a mindfulness-music therapy intervention to improve anxiety and stress in adolescents and young adults with cancer. J. Pain Symptom Manag. 63, e357–e363. doi: 10.1016/j.jpainsymman.2021.11.013, 34896280

[ref44] Koelsch S. (2014). Brain correlates of music-evoked emotions. Nat. Rev. Neurosci. 15, 170–180. doi: 10.1038/nrn3666, 24552785

[ref45] Konecni V. J. Wanic R. A. Brown A. (2007). Emotional and aesthetic antecedents and consequences of music-induced thrills. Am. J. Psychol. 120, 619–643. doi: 10.2307/20445428, 18277519

[ref46] Leder H. Belke B. Oeberst A. Augustin D. (2004). A model of aesthetic appreciation and aesth-etic judgments. Br. J. Psychol. 95, 489–508. doi: 10.1348/0007126042369811, 15527534

[ref47] Lee K. H. Wong D. T.-K. (2017). Chinese popular music as a musical heritage and cultural marker of the Malaysian Chinese. Int. J. Heritage Stud. 23, 989–1001. doi: 10.1080/13527258.2017.1362577

[ref48] Lin C. (2020). Relocating the functions of chineseness in Chinese popular music after the China wind. China Perspect. 2020, 7–14. doi: 10.4000/chinaperspectives.10068

[ref49] Liu X. Liu Y. Shi H. Li L. Zheng M. (2021a). Regulation of mindfulness-based music listening on negative emotions related to COVID-19: an ERP study. Int. J. Environ. Res. Public Health 18:7063. doi: 10.3390/ijerph18137063, 34280999 PMC8296951

[ref50] Liu X. Liu Y. Shi H. Zheng M. (2021b). Effects of mindfulness meditation on musical aesthetic emotion processing. Front. Psychol. 12:648062. doi: 10.3389/fpsyg.2021.648062, 34366968 PMC8334183

[ref51] Luo Q. Woramitmaitree N. Chuangprakhon S. (2025). The fusion of Chinese popular songs inspired by peking opera and western music genres in educational studies. Int. Educ. Stud. 18:108. doi: 10.5539/ies.v18n4p108

[ref52] Lutz J. Herwig U. Opialla S. Hittmeyer A. Jaencke L. Rufer M. . (2014). Mindfulness and emotion regulation – an fMRI study. Soc. Cogn. Affect. Neurosci. 9, 776–785. doi: 10.1093/scan/nst043 23563850 PMC4040090

[ref53] Menninghaus W. Wagner V. Schindler I. Hanich J. Jacobsen T. Koelsch S. (2019). What are aesthetic emotions? Psychol. Rev. 126, 171–195. doi: 10.1037/rev0000135, 30802122

[ref54] Meyer L. B. (2008). Emotion and Meaning in Music. Chicago: University of Chicago Press.

[ref55] Nieminen S. Istók E. Brattico E. Tervaniemi M. (2012). The development of the aesthetic experience of music: preference, emotions, and beauty. Music. Sci. 16, 372–391. doi: 10.1177/1029864912450454

[ref56] Nieminen S. Istók E. Brattico E. Tervaniemi M. Huotilainen M. (2011). The development of aesthetic responses to music and their underlying neural and psychological mechanisms. Cortex 47, 1138–1146. doi: 10.1016/j.cortex.2011.05.008, 21665202

[ref57] Nilsson U. (2008). The anxiety- and pain-reducing effects of music interventions: a systematic review. AORN J. 87, 780–807. doi: 10.1016/j.aorn.2007.09.013, 18395022

[ref58] North A. C. Hargreaves D. J. (1995). Subjective complexity, familiarity, and liking for popular music. Psychomusicol. J. Res. Music Cogn. 14, 77–93. doi: 10.1037/h0094090

[ref59] Palmer R. Roos C. Vafaie N. Kober H. (2023). The effect of ten versus twenty minutes of mindfulness meditation on state mindfulness and affect. Sci. Rep. 13:20646. doi: 10.1038/s41598-023-46578-y, 38001316 PMC10673854

[ref9004] Pereira C. S. Teixeira J. Figueiredo P. Xavier J. Castro S. L. Brattico E. (2011). Music and emotions in the brain: Familiarity matters. PLOS ONE, 6:e27241. doi: 10.1371/journal.pone.0027241 22110619 PMC3217963

[ref60] Rentfrow P. J. Gosling S. D. (2003). The do re mi’s of everyday life: the structure and personality correlates of music preferences. J. Pers. Soc. Psychol. 84, 1236–1256. doi: 10.1037/0022-3514.84.6.1236, 12793587

[ref61] Rodríguez-Carvajal R. (2014). Mindfulness and music: a promising subject of an unmapped field. Int. J. Behav. Res. Psychol. 4, 27–35. doi: 10.19070/2332-3000-140006

[ref62] Russell J. A. (2003). Core affect and the psychological construction of emotion. Psychol. Rev. 110, 145–172. doi: 10.1037/0033-295X.110.1.145, 12529060

[ref63] Sahdra B. K. MacLean K. A. Ferrer E. Shaver P. R. Rosenberg E. L. Jacobs T. L. . (2011). Enhanced response inhibition during intensive meditation training predicts improvements in self-reported adaptive socioemotional functioning. Emotion 11, 299–312. doi: 10.1037/a0022764, 21500899

[ref64] Salimpoor V. N. Benovoy M. Longo G. Cooperstock J. R. Zatorre R. J. (2009). The rewarding aspects of music listening are related to degree of emotional arousal. PLoS One 4:e7487. doi: 10.1371/journal.pone.0007487, 19834599 PMC2759002

[ref65] Sayers W. M. Creswell J. D. Taren A. (2015). “The emerging neurobiology of mindfulness and emotion processing” in Handbook of mindfulness and self-regulation. eds. Sayers W. M. Creswell J. D. Taren A. (New York: Springer), 9–22.

[ref70] Scherer K. Zentner M. (2008). Music evoked emotions are different–more often aesthetic than ut-ilitarian. Behav. Brain Sci. 31, 595–596. doi: 10.1017/S0140525X08005505

[ref66] Scherer K. R. (2004). Which emotions can be induced by music? What are the underlying mechanis-ms? And how can we measure them? J. New Music Res. 33, 239–251. doi: 10.1080/0929821042000317822

[ref67] Scherer K. R. (2005). What are emotions? And how can they be measured? Soc. Sci. Inf. 44, 695–729. doi: 10.1177/0539018405058216

[ref69] Scherer K. R. Zentner M. R. (2001). “Emotional effects of music: production rules” in Music and emotion. eds. Juslin P. N. Sloboda J. A. (New York, NY: Oxford University Press), 361–392.

[ref68] Scherer K. R. Schorr A. Johnstone T. (2001a). Appraisal processes in emotion: Theory, methods, research. Oxford: Oxford University Press.

[ref71] Scherer K. R. Zentner M. R. Schacht A. (2001b). Emotional states generated by music: an explo-ratory study of music experts. Music. Sci. 5, 149–171. doi: 10.1177/10298649020050S106

[ref72] Schindler I. Hosoya G. Menninghaus W. Beermann U. Wagner V. Eid M. . (2017). Measuring aesthetic emotions: a review of the literature and a new assessment tool. PLoS One 12:e0178899. doi: 10.1371/journal.pone.0178899, 28582467 PMC5459466

[ref73] Schubert E. (2007). The influence of emotion, locus of emotion and familiarity upon preference in music. Psychol. Music 35, 499–515. doi: 10.1177/0305735607072657

[ref74] Schubert E. (2013). Emotion felt by the listener and expressed by the music: literature review and theoretical perspectives. Front. Psychol. 4:837. doi: 10.3389/fpsyg.2013.00837, 24381565 PMC3865445

[ref75] Singh D. Suhas A. Naveen K. Nagendra H. (2014). Measures of mindfulness and anxiety in OM meditators and non-meditators: a cross-sectional study. Int. J. Med. Public Health 4:110. doi: 10.4103/2230-8598.127170

[ref76] Sleimen-Malkoun R. Devillers-Réolon L. Temprado J.-J. (2023). A single session of mindfulness meditation may acutely enhance cognitive performance regardless of meditation experience. PLoS One 18:e0282188. doi: 10.1371/journal.pone.0282188, 36920902 PMC10016675

[ref77] Somaraju L. H. Temple E. C. Bizo L. A. Cocks B. (2023). Brief mindfulness meditation: can it make a real difference? Curr. Psychol. 42, 5530–5542. doi: 10.1007/s12144-021-01897-z

[ref78] Swaminathan S. Schellenberg E. G. (2015). Current emotion research in music psychology. Emot. Rev. 7, 189–197. doi: 10.1177/1754073914558282

[ref79] Tan L. B. G. Lo B. C. Y. Macrae C. N. (2014). Brief mindfulness meditation improves mental state attribution and empathizing. PLoS One 9:e110510. doi: 10.1371/journal.pone.0110510, 25329321 PMC4201548

[ref80] Tong M. Ji C. (2024). The application and innovation of erhu music in modern popular music. Appl. Math. Nonlinear Sci. 9:20241664. doi: 10.2478/amns-2024-1664

[ref9007] Tracy J. L. Randles D. (2011). Four models of basic emotions: A review of Ekman and Cordaro, Izard, Levenson, and Panksepp and Watt. Emotion Review, 3, 397–405. doi: 10.1177/1754073911410747

[ref81] Trost W. Ethofer T. Zentner M. Vuilleumier P. (2012). Mapping aesthetic musical emotions in the brain. Cereb. Cortex 22, 2769–2783. doi: 10.1093/cercor/bhr353, 22178712 PMC3491764

[ref82] Watson D. Clark L. A. Tellegen A. (1988). Development and validation of brief measures of positive and negative affect: the PANAS scales. J. Pers. Soc. Psychol. 54, 1063–1070. doi: 10.1037/0022-3514.54.6.1063, 3397865

[ref84] Wolf R. (2019). In Tune: Music as the Bridge to Mindfulness. 1st Edn. New York: The Experiment LLC.

[ref85] Yan M. (2022). Peking opera pitched to younger audiences. Available online at: https://www.chinadaily.com.cn/a/202204/25/WS62697dcca310fd2b29e59ba4.html

[ref86] Young S. D. Kim J. Hanley A. (2025). Mindful jazz and preferred music interventions reduce pain among patients with chronic pain and anxiety: a pilot randomized controlled trial. Cureus. 17:e80485. doi: 10.7759/cureus.80485, 40225443 PMC11991751

[ref9008] Zatorre R. J. Salimpoor V. N. (2013). From perception to pleasure: Music and its neural substrates. Proceedings of the National Academy of Sciences, 110(Suppl. 2), 10430–10437. doi: 10.1073/pnas.1301228110 PMC3690607 23754373

[ref87] Zentner M. Grandjean D. Scherer K. R. (2008). Emotions evoked by the sound of music: char-acterization, classification, and measurement. Emotion 8, 494–521. doi: 10.1037/1528-3542.8.4.494, 18729581

